# Zika virus crosses an in vitro human blood brain barrier model

**DOI:** 10.1186/s12987-018-0100-y

**Published:** 2018-05-15

**Authors:** Judie B. Alimonti, Maria Ribecco-Lutkiewicz, Caroline Sodja, Anna Jezierski, Danica B. Stanimirovic, Qing Liu, Arsalan S. Haqqani, Wayne Conlan, Mahmud Bani-Yaghoub

**Affiliations:** 1 Human Health Therapeutics Research Center, National Research Council of Canada, 100 Sussex Dr., Ottawa, ON Canada; 2 Human Health Therapeutics Research Center, National Research Council of Canada, Bldg M54-1200 Montreal Rd., Ottawa, ON K1A 0R6 Canada

**Keywords:** Zika virus, Brain endothelial cells, Blood–brain barrier model, iPSC, AXL, Neural progenitors

## Abstract

Zika virus (ZIKV) is a flavivirus that is highly neurotropic causing congenital abnormalities and neurological damage to the central nervous systems (CNS). In this study, we used a human induced pluripotent stem cell (iPSC)-derived blood brain barrier (BBB) model to demonstrate that ZIKV can infect brain endothelial cells (i-BECs) without compromising the BBB barrier integrity or permeability. Although no disruption to the BBB was observed post-infection, ZIKV particles were released on the abluminal side of the BBB model and infected underlying iPSC-derived neural progenitor cells (i-NPs). AXL, a putative ZIKV cellular entry receptor, was also highly expressed in ZIKV-susceptible i-BEC and i-NPs. This iPSC-derived BBB model can help elucidate the mechanism by which ZIKV can infect BECs, cross the BBB and gain access to the CNS.

**Electronic supplementary material:**

The online version of this article (10.1186/s12987-018-0100-y) contains supplementary material, which is available to authorized users.

## Introduction

Zika virus (ZIKV), a mosquito-borne flavivirus, was generally perceived to cause mild infections in adults. However, the 2015–2016 ZIKV outbreak in Brazil has been associated with increased cases of microcephaly, Guillain-Barré syndrome and other neurological conditions [1, 2]. There is substantial evidence that ZIKV is neurotropic (reviewed in [3]) since it is routinely found in brains of microcephalic infants [4] and human brain organoids [5]. ZIKV also shows tropism for the central nervous system (CNS) of mice [6, 7]. Neonatal ZIKV infection leads to abnormal vascular density and diameter in the developing brain, resulting in a leaky blood–brain barrier (BBB) and massive neuronal death [8]. Recent evidence suggests that ZIKV is also neurotropic in adults [9, 10] raising the possibility that the intact BBB can be ‘permissive’ to ZIKV infection allowing access to brain cells. In this study, a novel in vitro stem-cell derived BBB model was used to evaluate the ability of a 2013 ZIKV isolate (Asian lineage) [11] to cross the BBB and infect human neural cells.

The BBB is a tightly sealed and specialized endothelial cell layer which prevents many drugs, particulates and microbes from entering the brain. BBB models in vitro have been used routinely to evaluate the mechanisms of passage of therapeutics and viruses across the BBB [12–15]. To overcome limitations inherent in human primary and immortalized brain endothelial cell (BEC) models, including low baseline transendothelial electrical resistance (TEER), discontinuous tight junctions and fast loss of the BBB phenotype in culture [16], BBB models differentiated from human stem cells have been developed [17]. Recently, we have described [18] a human BBB model based on a two-step differentiation protocol of induced pluripotent stem cells (iPSCs) derived from amniotic fluid cells into induced brain endothelial cells (i-BECs), as well as into neural progenitor cells (i-NPs) and mature neurons (i-Ns) (Additional file 1: Figure S1). This i-BEC model has been shown to recapitulate the salient molecular and functional features of the BBB, including the expression of BBB-enriched transporters and receptors, high transendothelial electrical resistance (TEER) and the ability to discriminate species-selective mechanisms of receptor-mediated antibody transport [18]. Here, we provide a demonstration of this model’s utility in evaluating viral invasion across the BBB coupled with viral infection of target brain cells.

## Results

**Fig. 1:**
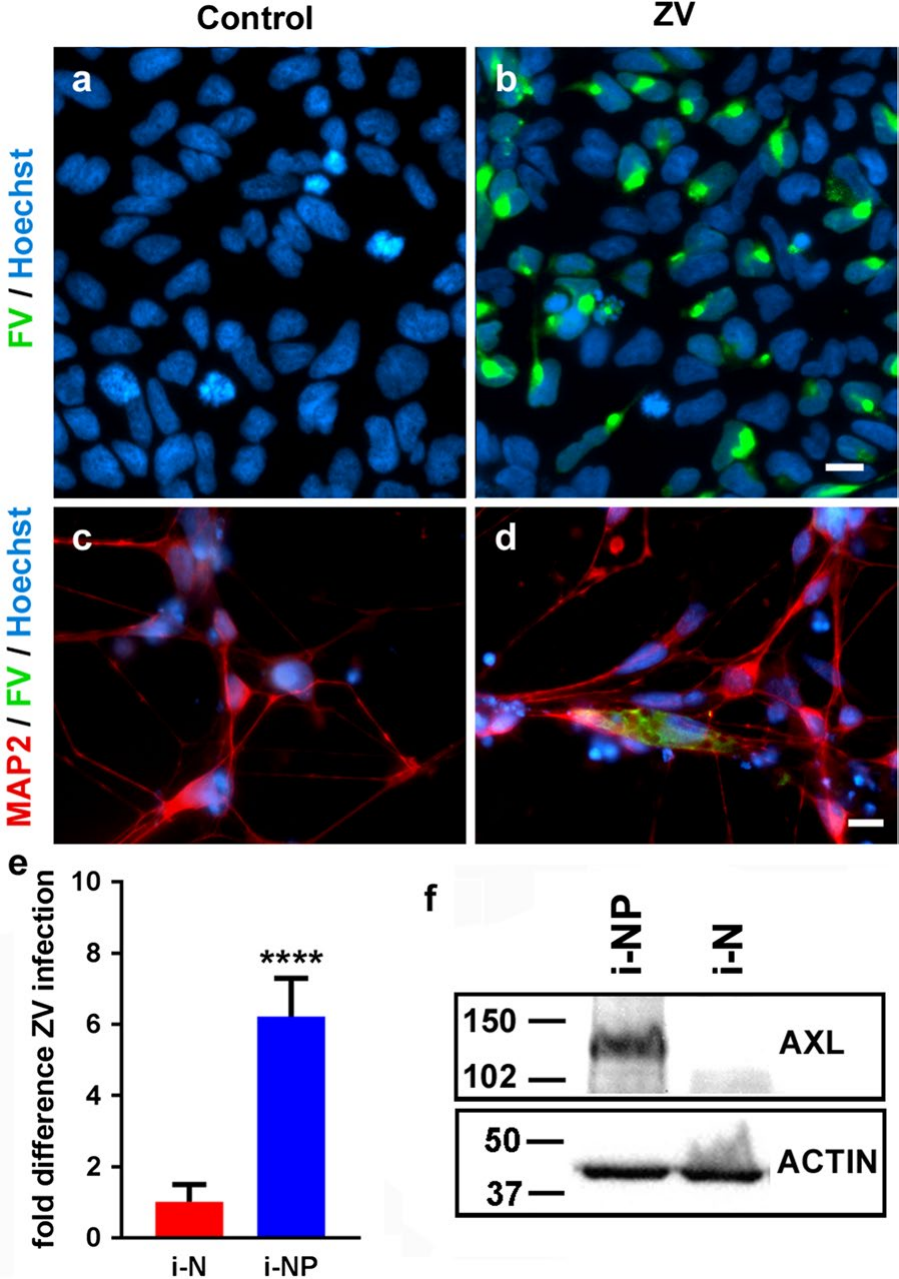
Direct ZIKV infection and AXL expression in i-NP and i-Ns. **a**–**d** ZIKV infection was detected in the cytoplasm of **b** i-NPs and **d** i-Ns by immunofluorescence staining 72 h after ZIKV infection (MOI = 4). The nuclei were stained with Hoechst dye (blue) and the virus was detected using anti-flavivirus group antibody (FV; green). i-Ns were additionally stained with the anti-MAP2 (red) antibody. Scale bar = 10 µm. **e** Fold difference of ZIKV (ZV) infection in i-NP, relative to i-Ns, based on cell counts of FV positive cells (mean ± SD). Cell counts are from two independent experiments, six fields counted, with at least 80 cells/field. (****p < 0.0001, Student’s T test). **f** A representative Western blot of putative ZIKV receptor AXL expression in i-NPs and i-Ns detected by an anti-AXL antibody. β-ACTIN was used as an internal loading control

Initially, to confirm that ZIKV could infect human i-NPs and i-Ns, the virus was added directly to these cells for up to 72 h (Fig. 1). i-NPs were highly susceptible (Fig. 1a, b), whereas terminally differentiated iNs were much less permissive to ZIKV infection (Fig. 1c, d; quantified in Fig. 1e). The receptor tyrosine kinase AXL, a putative entry receptor that mediates endocytosis of flaviviruses into host cells [19, 20] has been proposed as a candidate ZIKV entry receptor in human glial cells [21], neural stem cells [19] and radial glial cells [19]. The putative ZIKV receptor AXL was also highly expressed in the i-NPs but not in i-Ns (Fig. 1f), which correlated with the lower infection of i-Ns observed in this study (Fig. 1e) and as previously described [22, 23].

**Fig. 2:**
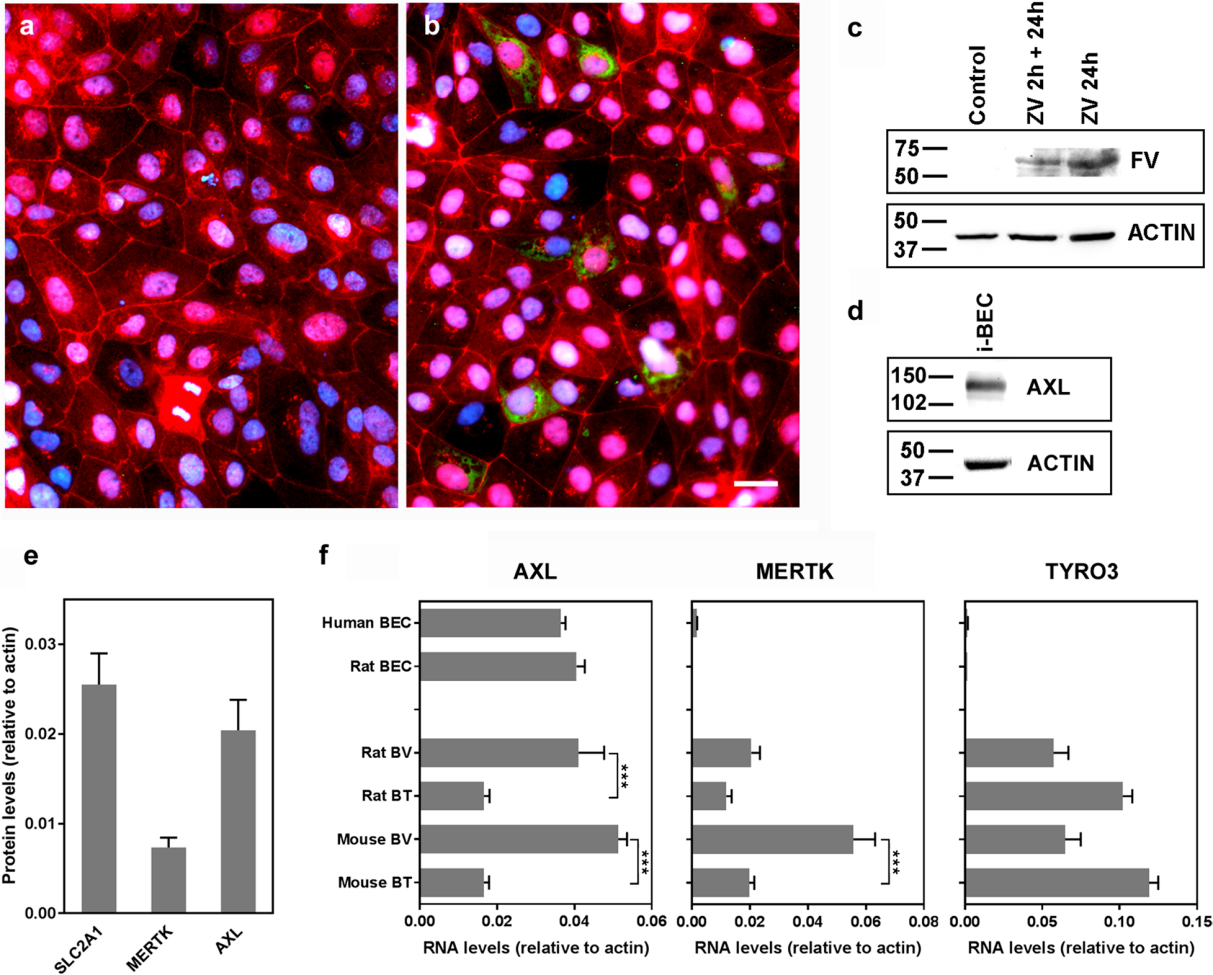
ZIKV infection and AXL expression in BECs. **a**, **b** Immunofluorescence staining was conducted 24 h post ZIKV infection (MOI = 4) of i-BECs using anti-flavivirus group antibody (FV; green). ZIKV was detected in infected i-BECs (**b**) but not in uninfected controls (**a**). Tight junction marker ZO-1 was used to label cell–cell contacts (red); nuclei were stained using Hoechst dye (blue). Scale bar = 10 µm. **c** The presence of ZIKV in i-BECs was also determined by Western blotting at 2 or 24 h after exposure to the virus. **d** Representative Western blot demonstrating putative ZIKV receptor AXL expression in i-BECs using an anti-AXL antibody. β-ACTIN was used as an internal loading control. **e** Expression levels of SLC2A1 (GLUT-1), MERTK and AXL in human brain endothelial cell line hCMEC/D3 determined by proteomics (LC–MS). Shown are ratio of intensity-based absolute quantification values (mean ± SD from 3 independent cell preparations) calculated for each protein and β-ACTIN, as described previously [41]. **f** The expression of *AXL*, *MERTK* and *TYRO3* in primary human and rat brain endothelial cells (BECs) or in isolated rat or mouse brain vessels (BV) or whole brain tissues (BT), determined by NGS. Shown are abundances (mean ± SD) of RNA transcripts relative to *β*-*ACTIN* from at least three cell/tissue preparations (***p < 0.001, One-Way ANOVA, Tukey’s post hoc test)

Similarly, ZIKV infection of the i-BECs was identified 24 h post-exposure to the virus by immunofluorescence (Fig. 2a, b) and was detected in cell lysates by Western blotting after only a 2-h exposure (Fig. 2c). ZIKV-infected i-BEC monolayers remained viable with no visible compromise to the integrity and continuity of their intercellular contacts, as assessed by their staining for a tight junction protein, ZO-1 (Fig. 2b). Putative ZIKV receptor AXL was highly expressed in i-BECs by Western blot analysis (Fig. 2d) as well as in human brain endothelial cell line hCMEC/D3 by LC–MS (Fig. 2e). In hCMEC/D3 cells, AXL expression levels were similar to those of SLC2A1 (GLUT1), and higher than the expression of two other putative ZIKV receptors MERTK and TYRO3 (which was not detectable) (Fig. 2e). *AXL* was also identified as a highly abundant transcript in primary human and rat brain endothelial cells by NGS analyses (Fig. 2f) and was highly enriched in isolated rat and mouse brain microvessels, compared to whole brain tissues (Fig. 2f). In contrast to low expression of *MERTK* and *TYRO3* transcripts in brain endothelial cells (Fig. 2e, f), both *MERKT* and *TYRO3* were expressed in isolated brain microvessels and total brain extracts (Fig. 2f). AXL-mediated infection by ZIKV has been shown in a variety of endothelial cells [24] including human umbilical vein endothelial cells (HUVECs) [25] and could play a role in ZIKV infection of brain endothelial cells, and specifically, i-BECs.

To determine whether ZIKV could cross the BBB, virus was added into the apical compartment of the Transwell inserts containing i-BEC monolayers. The inserts were then placed in a 12 well companion plate containing adherent i-NPs (Additional file 1: Figure S1). If ZIKV crossed the BBB, the virus would be present in the fluid compartment between the two cellular layers resulting in i-NP infection.

**Fig. 3:**
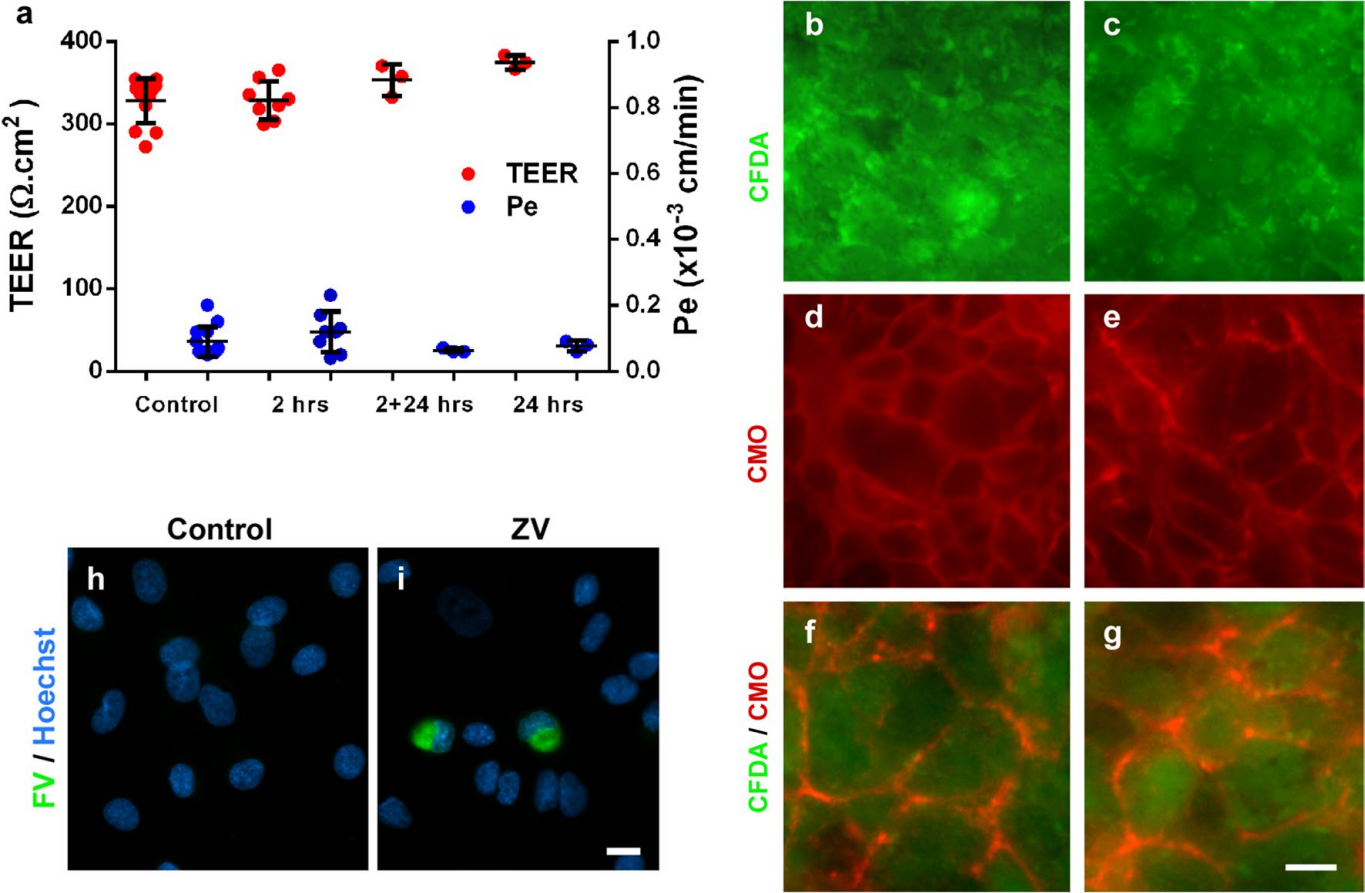
ZIKV crosses the human BBB and infects i-NPs. i-BECs were cultured on Transwell inserts and the cells were mock-infected (control) or infected with ZIKV. **a** TEER (Ω cm^2^) was measured on each i-BEC insert, to ensure barrier integrity was intact, before infection with ZIKV. Only i-BEC inserts with TEER values ≥ 300 Ω cm^2^ were used in the assay. Sodium fluorescein permeability (Pe; cm/min) were measured in infected i-BECs at 2 h (2 h), 2 h infection followed by 24 h recovery (2 + 24 h) or 24 h (24 h) post ZIKV-infection with no recovery (mean ± SD). Low sodium fluorescein impermeability (Pe) during the course of ZIKV infection, similar to mock-infected controls (ns, p > 0.1, One-way ANOVA), confirmed an intact barrier. To demonstrate i-BEC viability and continuity of membrane contacts, uninfected (**b**, **d**, **f**) and ZIKV-infected (**c**, **e**, **g**) i-BECs were co-labeled with CFDA (**b**, **c**; green) and CellMask Orange (CMO) (**d**, **e**; red); overlay images shown in (**f**–**g**), on the Transwell membranes at the end of ZIKV transport experiments (24 h). **h**, **i** ZIKV crossing of the BBB and infection of underlying i-NPs (**i**) was confirmed through immunofluorescence staining with an anti-flavivirus antibody (FV; green) 24 h post-infection. Scale bar = 20 µm

To assess BBB integrity in i-BECs during ZIKV exposure, two parameters were examined: TEER, prior to infection, and sodium fluorescein permeability post-infection. In this BBB model, TEER values have been shown to closely inversely correlate with sodium fluorescein paracellular permeability [18]. Following infection of the i-BECs, the BBB paracellular permeability remained unchanged, ascertained by sustained restriction of sodium fluorescein passage in infected (0.10 ± 0.05 × 10^−3^ cm/min) compared to mock-infected controls (0.09 ± 0.05 × 10^−3^ cm/min) (Fig. 3a). At the end of ZIKV exposure, the i-BECs in Transwells were instantly co-stained with the vital dye CFDA and the plasma membrane dye CellMask Orange (Fig. 3b–g) confirming cell viability and intactness of cell–cell contacts.

During the exposure of i-BEC cultured in Transwell inserts, ZIKV was able to cross the BBB, escape at the abluminal surface of i-BEC monolayers and infect i-NPs cultured in the companion wells below (Fig. 3h, i). Since the paracellular barrier integrity measured by sodium fluorescein permeability remained unchanged during this transmigration process, the experiment suggested that ZIKV did not cross the BBB through disrupted tight junctions. Similarly, the release of ZIKV internalized into i-BECs by cytolysis can be discounted since the viability of i-BECs was not affected following ZIKV infection (Fig. 3b, c).

## Discussion/conclusion

This report demonstrates that ZIKV can cross the BBB formed in vitro by iPSC-derived i-BEC, i-NP and i-Ns and subsequently infect susceptible neural cells. In line with these findings, two recent studies have shown that ZIKV infects, triggers inflammatory activation and releases basolaterally from primary human brain microvascular cells (hBMECs) [26] grown in Transwell inserts, without an overt barrier disruption [27]. The drawback of these studies was low (< 100 Ω cm^2^) baseline TEER of primary hBMECs, which could ‘mask’ subtle changes in paracellular tightness caused by harmful agents. In contrast, a 3D BBB model formed by a human BEC line grown in rotating bioreactor under flow conditions exhibited high resistance to infection by diverse RNA viruses, including ZIKV [12].

The current study confirms that ZIKV infects and crosses a Transwell human BBB model with TEER values ≥ 300 Ω cm^2^, without compromising its paracellular permeability for small molecule sodium fluorescein or the viability of the i-BEC monolayer. Based on these observations, we hypothesize that the primary mechanism of ZIKV traversing the BBB is through i-BEC infection, transcellular passage and release on the abluminal side of the monolayer. However, we cannot exclude the possibility that some viral particles could selectively modulate tight junctions and cross into the bottom chamber via paracellular diapedesis without overtly disrupting the BBB permeability; similar mechanism has been described for primed inflammatory T-cells [28]. Since ZIKV has been shown to induce inflammatory activation of brain endothelial cells [26], it might also affect other barrier properties of i-BECs, including transporter function. Several other mechanisms for neurotropic viruses to enter the CNS have been proposed [29, 30]. Notably, the ability of ZIKV to cross the placenta [31] via infected monocytes has also been demonstrated. Although transport via infected immune cells was not examined in this study, it remains a possible natural route of entry for ZIKV into the brain in vivo.

This study further demonstrates that the basolateral release of ZIKV from i-BEC monolayers results in the efficient infection of AXL-expressing i-NPs. Infection of i-Ns, in which AXL is down-regulated, was much lower. Susceptibility of NPs to ZIKV infection has previously been linked to AXL-mediated ZIKV entry [19, 32] leading to increased cell death, dysregulation of cell-cycle progression and attenuated growth [23]. Similar to i-NPs, ZIKV tropism for endothelial cells has also been shown to positively correlate with AXL expression [24, 25]. Since AXL ablation/inhibition in some studies did not result in reduced ZIKV infection [33, 34] other flavivirus adhesion and entry cofactors, including glycosaminoglycans, integrins and other phosphatidylserine (PS) receptors of the TIM (T cell immunoglobulin and mucin domain) and TAM (TYRO3, AXL, and MERTK) families [35], provide additional molecular context for ZIKV permissiveness of various neural cell types, and possibly BECs. In contrast to MERTK and TYRO3, AXL is highly abundant in human brain endothelial cells, including i-BECs, and is also enriched in brain microvessels compared to target brain tissue, potentially contributing to brain tropism of ZIKV. Since ZIKV infection of human BEC in this and other studies did not produce cytotoxic effects [24, 25, 27], it has been proposed that BECs could act as a viral reservoir enabling persistent ZIKV infection and replication, hindering its clearance from neuronal tissues [26].

This in vitro study demonstrates that ZIKV can cross the intact BBB, potentially via a transcellular pathway, and infect susceptible neural cells, notably i-NPs. Understanding the mechanisms of neurotropic virus-mediated BBB and CNS penetration will be important in designing strategies to reduce the devastating CNS sequelae of ZIKV infection. The human iPSC-derived BBB models, in conjunction with allogeneic i-NPs and i-Ns such as used in this study, provide an important advantage for studies of bacterial [36] and viral invasion into CNS, in particular for those pathogens that have poor infectivity in rodent models and high tropism for neural cells. The model used here represents a valuable tool to aid in the development and screening of antiviral therapeutics to limit ZIKV and other viral-induced pathologies in the CNS.

## Methods

### ZIKV strain and culturing

Zika virus (ZIKV) was isolated in 2013 from a Canadian patient who had recently returned from Thailand [11]. The ZIKV belongs to the Asian lineage and was a gift from Dr. D. Safronetz at the National Microbiology Laboratory, Winnipeg, Manitoba. ZIKV was grown in Vero cells (ATCC CCL-81.5) with DMEM, 10% heat inactivated fetal bovine serum (HI FBS), 100 U/ml penicillin and 100 µg/ml streptomycin (Pen/Strep). The cell culture supernatant was centrifuged at 1200×*g*, 10 min, 4 °C to remove cell debris, then concentrated with a 2 h centrifugation at 30,000×*g*, 4 °C. The pellet was re-suspended in a small volume of the supernatant plus 10% HI FBS final. ZIKV was titrated on Vero E6 cells.

### Differentiation of iPSCs into i-BEC, i-NPs and i-Ns

All studies with human cells and tissues have been approved by the Ottawa Hospital- and the National Research Council of Canada’s Research Ethics Boards. Amniotic fluid cells (AFCs) were obtained under informed written consent at 26 weeks of gestation from a single female following routine amniocentesis. Genetic analysis revealed normal karyotypes. The AFC were cultured in DMEM supplemented with 20% FBS, as previously described [37]. Induced pluripotent stem cells (iPSCs) were generated by transfecting AFCs with oriP/EBNA1 episomal vectors EP4 E02S EN2K (Addgene-20925), pCEP4 M2L (Addgene-20926) and pEP4 E02S ET2K (Addgene-20927) using the Nucleofector I Device (Amaxa), as previously described [18]. To obtain iPSC-derived induced brain endothelial cells (i-BECs), a two-step differentiation protocol was applied, as previously described [18]. In brief, iPSCs were cultured in KnockOut DMEM/F12 medium (Life Technologies) supplemented with 20% KnockOut Serum Replacement, 1× Non-Essential Amino Acids, 1× Glutamax and 55 µM β-mercaptoethanol (all from Life Technologies) for 5–7 days after which the medium was switched to complete endothelial differentiation medium (EM) composed of human serum free endothelial medium (Life Technologies) supplemented with 1% platelet-poor plasma derived serum (PDS, Alfa-Aesar) and 20 ng/ml of bFGF (Life Technologies) for 9–10 days.

To generate iPSC derived neural progenitor cells (i-NPs), iPSCs were cultured in Matrigel coated plates in Stemdiff Neural Induction Medium (Stem Cell Technologies), according to manufacturer’s instructions and maintained for an additional 3 weeks with daily changes in the Stemdiff Neural Progenitor medium (Stem Cell Technologies). Neurons (i-Ns) were differentiated from i-NP monolayers in DMEM/F12 supplemented with B27 (both from Life Technologies) with medium replenished every other day. Detailed protocols for iPSC derived i-NP and i-N differentiation and characterization have been previously described in [18].

### Zika virus infection of i-BEC, i-NP, and i-Ns

For the direct infection of i-NP and i-Ns, the virus was added to the culture medium of the cells in a 12 well plate for 24–72 h at an MOI = 4. The mock-infected controls received the equivalent volume of DMEM, 10% HI FBS and Pen/Strep without ZIKV. The infection of the i-BECs with ZIKV was conducted 5 separate times at an MOI = 4 and samples were collected for analyses at 2 or 24 h post-infection. The immunofluorescence staining to detect ZIKV in infected cells performed in duplicate, in at least two separate experiments. The Western blot detection of ZIKV in cell lysates was conducted in two separate experiments.

### Immunofluorescence staining

i-NPs or i-Ns grown on glass coverslips were fixed for 10 min with 4% paraformaldehyde, washed with PBS and permeabilized for 20 min with 0.1% Triton X-100 in PBS. Cells were washed with PBS, incubated with protein block (DAKO) for 20 min, then with primary antibodies, diluted in antibody diluent (DAKO) for 1 h. Primary antibodies were: mouse monoclonal anti-flavivirus group antigen, (Cat # MAB10216 Millipore), rabbit monoclonal anti-AXL (Cell Signaling Cat#8661), mouse monoclonal anti-MAP2 (Sigma Cat #M1406) and rabbit polyclonal anti-ZO-1 (Invitrogen Cat#402200). Cells were washed with PBS, before adding secondary antibodies for 1 h. Secondary antibodies were ALEXA488 conjugated goat anti-rabbit IgG (Invitrogen Cat#A11008), ALEXA488-conjugated goat anti-mouse IgG (Life Technologies A21131), Rhodamine-conjugated goat anti-rabbit IgG (Invitrogen Cat#R6394), and Rhodamine-conjugated goat anti-mouse IgG (Invitrogen Cat#R6393). Cells were washed with PBS and mounted with fluorescent mounting medium (DAKO) spiked with 5 µg/ml of Hoechst 33258. Samples were imaged on a Zeiss Axiovert fluorescence microscope with an Axiocam HRm camera using a 20×/0.5 Plan Neofluar objective and Zeiss filter set 1 (488010-0000-000), EX BP 365/12, EM LP 397 for Hoechst, EM BP 515/65 for FITC, and EX BP 546/12, EM LP 590 for TRITC. Images were analyzed with Adobe Photoshop 7.0.1. The images obtained were done in duplicate and were repeated at least once.

### Western blotting

Cells were lysed in RIPA buffer (50 mM Tris, pH 7.4, 150 mM NaCl, 2 mM EDTA, 0.1% SDS, 1% Deoxycholate, 1% triton X-100, 1× protease inhibitor (Roche) and boiled in a water bath for 5 min to inactivate virus. Proteins were separated by 10% SDS-PAGE and transferred to nitrocellulose. Membranes were probed with rabbit monoclonal anti-AXL (Cell Signalling), or mouse monoclonal anti-flavivirus antibodies (Millipore) and anti-ACTIN (BioRad) followed by HRP-conjugated secondary antibodies and were developed with Clarity ECL kit (Biorad) and imaged on a Fluorochem Q imager (Alpha Innotech). Images were analyzed in Adobe Photoshop and original scanned images are shown in Additional file 1: Figure S2.

### BBB model in vitro using transwell culture system

The human i-BEC transwell BBB model was generated, as previously described [18]. Briefly, the i-BECs were dissociated with Accutase (Stem Cell Technologies) and seeded at a density of 5 × 10^5^ i-BECs per 0.9 cm^2^ cell growth area on a Transwell insert (1 µm pore size, BD-Falcon) coated with 0.5% gelatin in 1 ml complete EM medium with 10 µM Y27362 (ROCK Inhibitor, Stem Cell Technologies); 2 ml of the same medium were added to the bottom (basal) wells of the companion plate, and incubated overnight at 37 °C in 5% CO_2_. The following day, the complete EM medium, without the Rock Inhibitor, was replaced only in the apical compartment of the Transwell insert and confluent monolayer formation was assessed 48 h post-plating.

### BBB in vitro permeability assays

Prior to being used in the BBB permeability assays, each i-BEC insert was assessed by measuring transendothelial electrical resistance (TEER). TEER measurements were conducted on a CellZscope apparatus (Nanoanalytics) using 1 cm diameter electrodes and standard spectrum settings: frequency 1 Hz–100 kHz, points per decade 9 and logarithmic spacing. Final TEER values in Ω cm^2^ were calculated by subtracting TEER values for the empty inserts from the TEER values for the inserts with i-BECs. The i-BEC monolayers on each insert were considered intact when the TEER values were ≥ 300 Ω cm^2^, as previously described [18].

To assess the ability of ZIKV to cross the BBB in vitro the virus was added to the media in the apical Transwell insert at an MOI = 4. The inoculum was left on the cells for 24 h except for the experiment in which the inoculum was removed after 2 h and replaced with complete endothelial medium for additional 24 h (2 + 24 h). The inserts were placed in a 12 well companion plate containing i-NPs growing on the bottom of the well or on glass coverslips, as appropriate.

To test whether the permeability of the i-BEC monolayer was affected by ZIKV, sodium fluorescein permeability (Pe) (apical to basal) was also determined at various time points during the BBB exposure to ZIKV. The assay was conducted twice at 2 h, once at 2 + 24 h recovery and once at 24 h. Briefly, the i-BEC transwell inserts were washed sequentially in three consecutive wells containing 2 ml pre-warmed Hanks Balanced Salt Solution (HBSS) to remove residual medium. The inserts were then placed into companion plates containing 2 ml of pre-warmed transport buffer (5 mM MgCl_2_ and 10 mM HEPES in HBSS, pH 7.4), equilibrated to 37 °C for 5–10 min and then 500 µl of the transport buffer was carefully removed from the top (apical) chamber of each insert and replaced with 500 µl of sodium fluorescein (50 µg/ml) in transport buffer. The plates were then incubated at 37 °C with gentle rotation (20 rpm/min), and 100 µl of transport buffer was collected from the bottom (basal) companion wells at 15, 30, 45 and 60 min intervals for permeability analysis, as previously described [18]. Following each collection, 100 µl pre-warmed transport buffer were added back to the companion wells and the plates were returned to the incubator. Inserts without cells were used for the background controls. The quantitation of sodium fluorescein in each sample was measured using a fluorescent plate reader (excitation 485 nm and excitation 530 nm) and plotted against a standard curve (0–50 ng sodium fluorescein solution in transport buffer).

In addition, at the end of ZIKV exposure, i-BEC viability was assessed using 2.4 µg/ml CFDA staining (Invitrogen) and continuity of inter-cellular contacts was assessed using a plasma membrane stain, CellMask Orange (CMO, 5 µg/ml) (Life Technologies), as previously described [18]. The cells were imaged using a 20 × HMC objective on an Axiovert 200 M Microscope (Zeiss).

### Quantitation of AXL expression in brain endothelial cells and isolated vessels

All studies with human cells and tissues have been approved by the National Research Council’s Research Ethics Board. Three sources of primary human brain microvascular endothelial cells (hBMECs) were used: one generated in-house from normal brain tissue removed during surgery for epilepsy; Cell Systems (Cat# ACBRI 376, mycoplasma negative); and Angio-Proteomie (Cat #cAP-0002, mycoplasma negative). The HBMECs were pooled for further analysis. Adult rat immortalized brain endothelial cells were developed in house, as previously described [38]. The brain microvessels were isolated from rat or mouse brain tissues by sequentially passing tissue homogenates through 350, 112 and 20 µm Nitex filters. The microvessels were collected from the filter-top of both 112 and 20 µm filters and the flow-through was collected as the brain parenchyma fraction.

RNA was extracted from cultured HBMECs, as well as from brain microvessels and total brain tissues from three rats and mice using RNeasy Plus Mini kit (Qiagen) and NucleoSpin RNA plus kit (Macherey–Nagel GmbH & Co. KG), respectively, according to manufacturer’s instructions. Genomic DNA contamination was removed by Turbo DNA-Free Kit (Life Technologies). RNA quality was assessed using Agilent Bioanalyzer 2100. RNA-Seq Libraries were generated using the TruSeq RNA kit (Illumina), as per manufacturer’s instructions. The RNA-Seq libraries were quantified by qPCR according to the Illumina *Sequencing Library qPCR Quantification Guide* and the quality of the libraries was evaluated on Agilent Bioanalyzer 2100 using the Agilent DNA-100 chip. The RNA-Seq library sequencing was performed using Illumina Next-Seq. RNA-Seq data was analyzed using Galaxy (useGalaxy.org).

The protein quantification was performed as follows. Membranes of cultured human brain endothelial cell line, hCMEC/D3 (obtained from Dr. Pierre-Olivier Couraud, Institute Cochin, INSERM, Paris, France), were isolated as described in [39] and protein samples prepared and analysed by nano-LC–MS/MS exactly as described in [40]. For LC–MS measurements, intensity-based absolute quantification values were calculated for AXL and β-actin, as described previously [41]. The collection of transcript and proteome data was analyzed to determine the relative abundance of AXL in specific data sets compared to the whole transcriptome or proteome.

## Additional file

**Additional file 1.** Supplementary information.
