# Supplementary material for: Zika virus crosses an in vitro human blood brain barrier model
Source: Fluids Barriers CNS. 2018 May 15;15:15. doi: 10.1186/s12987-018-0100-y (PMC5952854; doi:10.1186/s12987-018-0100-y)
Supplement: Supplementary file 1 — Additional file 1. Supplementary information. [file 12987_2018_100_MOESM1_ESM.docx]

**Zika Virus Crosses an *In Vitro* Human Blood Brain Barrier Model**

Judie B. Alimonti^1^, Maria Ribecco-Lutkiewicz^2^, Caroline Sodja^2^, Anna Jezierski^2^, Danica B. Stanimirovic^2^, Qing Liu^2^, Arsalan S. Haqqani^1^, Wayne Conlan^1^ and Mahmud Bani-Yaghoub^2^

Human Health and Therapeutics Portfolio, National Research Council of Canada, 100 Sussex Dr.^1^ or Bldg M54-1200 Montreal Rd.^2^, Ottawa, Ontario, Canada

Corresponding Author

**Dr. Anna Jezierski**

Human Health and Therapeutics Portfolio

National Research Council of Canada

1200 Montreal Road

Ottawa, Ontario, K1A 0R6

[anna.jezierski@nrc.ca](mailto:anna.jezierski@nrc.ca)

Email addresses:

[judie.alimoti@nrc.ca](mailto:judie.alimoti@nrc.ca)

[maria.ribecco-lutkiewicz@nrc.ca](mailto:maria.ribecco-lutkiewicz@nrc.ca)

[caroline.sodja@nrc.ca](mailto:caroline.sodja@nrc.ca)

[danica.stanimirovic@nrc.ca](mailto:danica.stanimirovic@nrc.ca)

[qing.liu@nrc.ca](mailto:qing.liu@nrc.ca)

arsalan.haqqani@nrc.ca

[wayne.conlan@nrc.ca](mailto:wayne.conlan@nrc.ca)

mahmud.bani-yaghoub@nrc.ca

**Additional Figures**

Figure S1


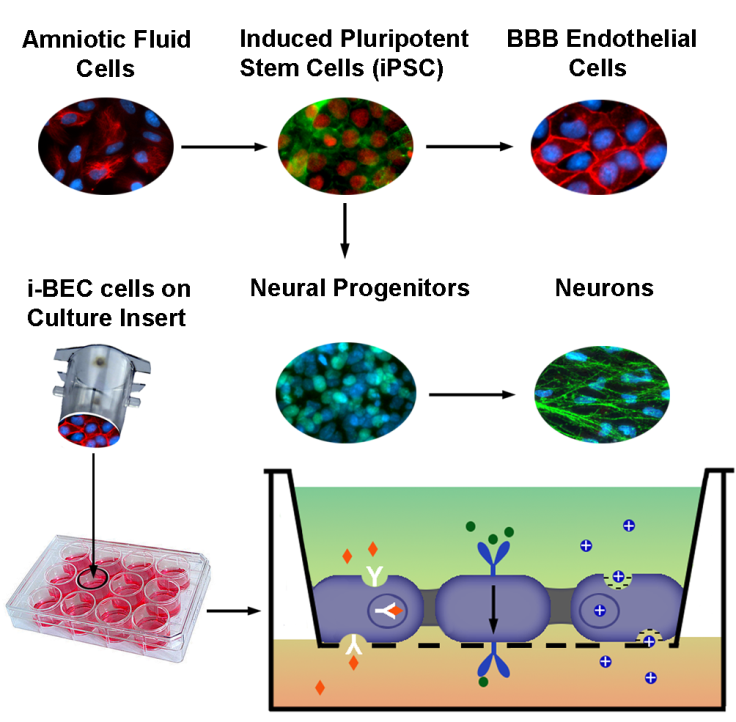


**Figure S1**: *Schematic of the* *iPSC-derived Blood-brain barrier (BBB) Transwell model.* Human brain endothelial cells (iBECs) that make up the BBB, as well as neural progenitor cells (iNPs) and neurons (iNs), were differentiated from amniotic fluid cell derived induced pluripotent stem cells (iPSCs). The iBECs were cultured on Transwell inserts to form an intact, impermeable BBB monolayer. The inserts were then placed into a 12-well companion plate containing iNPs. ZIKV permeability across the iBEC monolayer was assessed by adding virus into the upper (apical/luminal) compartment of the Transwell insert. If ZIKV was able to cross the BBB, than virus would be present in the bottom (basal/abluminal) fluid compartment between the two cellular compartments enabling infection of the iNPs in the companion plate.

Figure S2





**Figure S2**: *Original Western blot scan.*

Original scan of Western blots for anti-flavivirus (Millipore), anti-AXL (Cell Signaling) and anti ACTIN (BioRad) followed by HRP-conjugated secondary antibodies.
